# Amaranth as a Dual-Use Crop for Leafy Greens and Seeds: Stable Responses to Leaf Harvest Across Genotypes and Environments

**DOI:** 10.3389/fpls.2019.00817

**Published:** 2019-06-26

**Authors:** Natalie Hoidal, Maria Díaz Gallardo, Sven-Erik Jacobsen, Gabriela Alandia

**Affiliations:** ^1^Department of Plant and Environmental Sciences, University of Copenhagen, Copenhagen, Denmark; ^2^Instituto Pensamiento y Cultura en América Latina, Universidad Comunal Intercultural de Cempoaltépetl Barrio el Calvario, Tlahuitoltepec Mixe Oaxaca, Mexico; ^3^Quinoa Quality, Regstrup, Denmark

**Keywords:** plant-based protein, defoliation, nutrition, small farms, leafy green

## Abstract

Dual-use production systems that utilize the green leaves as well as seeds from amaranth are highly promising for small-scale farmers around the world. The leaves are an important source of nutrients for farming families, while seeds can provide income. Farmers who use amaranth as a dual-use crop are concerned about the impacts of defoliation on seed yield. This experiment tested defoliation at various intensities and frequencies (0, 25, 50, 75, and 100% defoliation, 1, 2, and 3 times) under controlled conditions as well as under Danish and Mexican field conditions. Defoliation tolerance was tested in a total of seven varieties, spanning the three primary grain amaranth species: *A. cruentus*, *A. hypocondriacus*, and *A. caudatus*. In all of the varieties and environments tested, we found that neither seed yield nor quality was impacted by a single defoliation event at intensities up to 50% leaf removal. We observed similar responses with two and three consecutive defoliations in which we removed 25% of all leaves. Greater frequency and intensity of defoliation resulted in reduced seed yield in some environments, while seed quality (protein content and 1000 KW) did not appear to be affected. Dual-use production systems should be promoted with small-scale farmers around the world as promising systems for improving local nutrition while maintaining profits from seed production. This paper provides baseline guidelines for farmers regarding optimal defoliation intensities and frequencies.

## Introduction

Amaranth, a crop that once sustained empires but disappeared from cultivation for centuries, is emerging once again and showing great potential for food and nutritional security around the world (Das, 2016). Due to its ability to be utilized both as a leafy green vegetable and as a grain, and its ability to grow in adverse conditions, amaranth can provide high quality nutrition in a wide array of contexts (Das, 2016). Most scientists agree that the origin of diversity for amaranth is southwestern Mexico near Oaxaca (Villarreal and Iturriaga, 2016). Due to its nutritional benefits, economic potential, and historical relevance to the region, organizations like Puente a la Salud Comunitaria have begun to promote amaranth cultivation in Oaxaca. Poverty rates in Oaxaca are amongst the highest in Mexico, with an estimated 70.4% of the population living in poverty in 2016^1^. Today, few farmers grow amaranth, but those who do receive high prices for the seed. In 2015, the authors of this paper asked farmers in the Valles Centrales (Central Valley) region of Oaxaca what prices they could get for maize vs. amaranth. Farmers reported amaranth seed yield similar to that of maize (∼0.7 to 1.2 t/ha), and they received approximately 25 pesos/kg of amaranth compared to 3 pesos/kg of maize. As a result of efforts by non-profit organizations and due to the cultural relevance and high prices of grain amaranth, the number of amaranth growers is increasing annually^2^.

This paper builds upon questions raised by Mexican farmers in 2015 about optimizing leaf harvest methods so they could benefit nutritionally from their crops while securing a good seed yield to contribute to their livelihoods. Farmers in the region use various cultivars of *A. cruentus* and *A. hypocondriacus*, which are typically thought of as grain species. If seed yield were not significantly impacted by leaf harvest in these species, this would represent a useful way for farmers to benefit both nutritionally and economically from their crops. A 2014 (unpublished) survey administered by Puente a la Salud Comunitaria with 118 farmer respondents from the Oaxaca region found that 86% of respondents harvested leaves and seeds from the same amaranth plants. Of these farmers, 97% of them harvested some leaves for family consumption, whereas only 10% sold the leaves they harvested. In discussions with the authors of this paper, farmers expressed apprehension about harvesting too many leaves for fear of decreasing their seed yield.

Dinssa et al. (2018) strengthened the case for the need for more research on dual-use species of amaranth, noting that small-scale farmers in sub-Saharan Africa would benefit from amaranth plants that could provide quality nutrition from both leaves and seeds. In their study, they focused on *A. cruentus* and *A. hypocondriacus*.

The use of amaranth as a dual-use crop for seed and leaf harvest has been documented from pre-colonization Mesoamerica; the 16th century Codex of Florentine, an ethnographic report by a Franciscan friar, contains images of indigenous farmers using both the leaves and seeds of amaranth (Das, 2016). Various studies have suggested that grain amaranths are highly tolerant to defoliation (Moreno et al., 1999; Castrillón-Arbeláez et al., 2012; Vargas-Ortiz et al., 2013, 2015). In some areas of Mexico, farmers actively remove 10–40% of the primary shoot biomass to enhance secondary branching and biomass productivity (Castrillón-Arbeláez et al., 2012).

The few studies that have investigated leaf harvest in amaranth have focused primarily on response mechanisms for tolerance to defoliation and identification of promising genotypes for dual-use production. Following extensive defoliation, Vargas-Ortiz et al. (2013) found that root growth was arrested in amaranth plants following defoliation and that plants compensated by using carbohydrate reserves from their stems and roots to assist with re-growth. The plants in their study also reduced stem and root sucrose synthase and cell wall invertase production, signaling a shift in resources from sink to source tissues (Vargas-Ortiz et al., 2013). A second study by Vargas-Ortiz et al. (2015) found that defoliation is followed by an altered transcription of genes coding for sucrolytic enzymes and remobilization of carbon from the stem and roots (Vargas-Ortiz et al., 2015).

Papers by Roitner-Schobesberger and Kaul (2013) and Dinssa et al. (2018) investigated the impact of 100% defoliation on amaranth seed yield. While this information provides useful insight on mechanisms for tolerance and genotypes that may be promising for dual-use production, it does not provide directly translatable information for farmers about the degree of leaf harvest that could be harvested before seed yield is impacted. While Roitner-Schobesberger and Kaul (2013) and Dinssa et al. (2018) identified genetic differences in tolerance to defoliation between cultivars at 100% defoliation, we sought to know whether these genetic differences would still manifest in seed yield differences with less intensive defoliation treatments, and whether these differences would be environment-dependent.

Three experiments have provided valuable information on low to moderate intensity defoliation effects in amaranth. Vargas-Ortiz et al. (2013) measured the impacts of 20, 50, and 100% defoliation in greenhouse conditions, and 50 and 100% defoliation field conditions in Celaya, Guanajuato, Mexico. These studies were done with amaranth varieties from Mexico. Moreno et al. (1999) examined the impact of various levels of low to moderate defoliation (10 and 40%) in field conditions in Mexico, but they did not examine the impacts on yield. Roitner-Schobesberger and Kaul (2013) examined the impact of 100 and 50% defoliation on seed yield in amaranth, but defoliation was performed during anthesis, rather than the vegetative stage, and only one variety at the moderate defoliation level (50%). Since at 100% defoliation Roitner-Schobesberger and Kaul (2013) found a significant genotype x environment interaction, it’s important to test their findings about more moderate defoliation treatments with more genotypes. While they found no seed yield impact following the 50% defoliation event, this result was from a specific climate with only one variety, and thus replication is necessary before broad recommendations can be drawn from this result. In all of these papers, defoliation occurred only once.

This study builds on the work of these authors by investigating the degree to which tolerance to defoliation is widespread across amaranth species, cultivars, and environments. By testing more moderate defoliation treatments (25, 50, and 75% defoliation in addition to 100%) at different frequencies (a single harvest vs. 2 or 3 consecutive harvests) we aimed to determine whether baseline recommendations on leaf harvest practices could be developed across genotypes. These treatments were studied in multiple environments, including a rain-fed system in Oaxaca, Mexico, which has a substantially drier climate than those studied in Moreno et al. (1999) and Vargas-Ortiz et al. (2013). We tried these experiments on a different set of cultivars; the varieties studied in most of the previous experiments all came from Mexico, where plants have been used for dual-use purposes for hundreds of years. By testing defoliation tolerance in a set of cultivars from Europe alongside Mexican cultivars, we aimed to determine whether defoliation tolerance is common across many genotypes, or only those used in Central America. We also explored the relationship between harvest intensity (% defoliation) and harvest frequency.

We hypothesized that dual-use production systems could be viable across different environments and genotypes. To test this hypothesis, we performed defoliation experiments with seven varieties of amaranth spanning the three main grain amaranth species, and tested defoliation tolerance in three environments: controlled growth chamber conditions, Danish (Sjælland) field conditions, and Mexican (Oaxaca) field conditions.

## Materials and Methods

### Study Sites

#### Growth Chamber Experiment

This study began in 2015 with a preliminary growth chamber experiment at the experimental station of Højbakkegaard, Taastrup, Faculty of Science, University of Copenhagen. The purpose of this trial was to gain baseline data on the impact of defoliation in amaranth under controlled conditions. The temperature was set to 22 ± 2°C during the day, and 13 ± 2°C during the night, with 12 h day lengths. Irradiance in the growth chamber was measured with a Li-Cor quantum sensor model LI-250, with an average of 573 μmol m^–2^, s^–1^ with the lights on. This corresponded to a daily average of 24.7 mol m^–2^ d^–1^. Pots with 25 cm height and 16 cm diameter were filled with 5 kg of Pindstrup potting mix (pH 5.5–6.5, N content 182 g/m). Four amaranth seedlings were planted per pot, thinned to one plant after 2 weeks and watered with 475 ml water per day. The water contained a standard nutrient solution (10 kg/100 L water) of Pioneer Makro (NPK 14-3-23 + Mg) and 1 L/100 L water Pioneer Mikro (B 0.23%, Cu 0.14%, chelated-iron DTPA/EDTA 1.32%, Mn 0.50%, Mo 0.05%, Zn 0.18%).

#### Field Experiments

Two Danish field trials took place in 2016 and 2017 at the experimental station of Højbakkegaard, Taastrup, part of the Faculty of Science, University of Copenhagen (55° 40′ 9″ N, 12° 18′ 35″ E, 28 m above sea level). An additional Mexican field trial took place in 2016 in the production fields of a local amaranth producer in the community of Santiago Suchilquitongo Etla, Oaxaca (17.25°N, 96.87°W, 2240 meters above sea level). This grower was part of a growers association based in Etla, Oaxaca (Central Valleys region). Conditions for each experiment including sowing date, soil type, sowing density, watering and fertilization regimes, crop rotation, sowing density, and pesticide use are reported in Table 1. Unfortunately, soil testing was not available for the field trial in Oaxaca, so a rough estimate of soil type is provided in Table 1. Weather conditions for both locations during treatment years are reported in Table 2; Danish weather data was obtained from the weather station at the experimental field station, and Mexican weather data is reported as average data for Oaxaca.

**TABLE 1:** Field trial conditions for Danish and Mexican field trials.

**Study site**	**Højbakkegaard, Taastrup**	**Højbakkegaard, Taastrup**	**Santiago Suchilquitongo Etla, Oaxaca**
Sowing date	20/05/2016	15/05/2017	26/08/2016
Soil type	J6 – sandy clay loam, pH 6.5, organic matter 2.2%	J6 – sandy clay loam, pH 6.6, organic matter 2.2%	Dystric and calcic cambisol
Sowing density	1 kg/ha	1 kg/ha	Hand-sown, 30 cm between rows, 10 cm within rows
Prior crop	Spring Barley	Spring Barley	Milpa^*^
Fertilizer	80 kg/ha NS 26 5 (26% N, 5% S) granular application on May 31	80 kg/ha NS 26 5 (26% N, 5% S) granular application on May 31	Applied by hand; 150 g of composted cow manure at the base of each plant 15 DAS
Pesticide use	None	Pirimor G (Certis) at 0.15 kg/ha on June 28 for flea beetles	None
Irrigation	None; rain-fed, see Table 2 for precipitation data	None; rain-fed, see Table 2 for precipitation data	None; rain-fed, see Table 2 for precipitation data

^*^Milpa is a commonly used farming system in Mexico that consists primarily of maize, and includes a variety of intercropped plants potentially including squash, beans, amaranth, and other leafy greens.

**TABLE 2:** Average monthly temperatures (Temp) and total monthly precipitation (Precip) during field trials in Taastrup, Denmark and Oaxaca, Mexico in 2016 and 2017.

	**Denmark 2017**		**Denmark 2016**		**Mexico 2016**	
	**Temp (°C)**	**Precip (mm)**	**Temp (°C)**	**Precip (mm)**	**Temp (°C)**	**Precip (mm)**
January	0.97	24.36	0.07	44.32	19.5	0.00
February	1.91	56.20	2.48	55.86	19.2	n.a.
March	4.86	55.11	3.56	49.58	22.4	3.0
April	6.62	65.42	6.79	59.58	24.6	0.0
May	12.41	27.27	13.65	30.33	25.7	76.4
June	15.41	92.73	16.64	59.69	22.6	178.0
July	16.05	94.46	17.22	96.67	22.3	145.1
August	16.63	73.94	16.53	66.10	22.6	59.1
September	13.58	154.27	16.23	24.11	22.4	64.5
October	11.08	79.54	8.90	90.58	21.4	8.5
November	5.71	71.68	4.09	59.58	20.2	4.1
December	3.49	45.79	4.41	29.37	20.2	0.0

*Gray shading correspond to months in which the experiments were conducted. Sources: Danish data – University of Copenhagen’s weather station in Taastrup, Denmark. Mexican data – Weather Online https://www.weatheronline.mx/weather/maps/city?LANG=mx&PLZ=_____&PLZN=_____&WMO=76775&CONT=mxmx&R=0&LEVEL=162&REGION=0020&LAND=MX&MOD=tab&ART=TEM&NOREGION=0&FMM=1&FYY=2016&LMM=12&LYY=2016. Oaxaca data are reported from the Oaxaca International Airport, which is 27 km away from the village of Etla, thus reported weather data is only approximate.*

### Experimental Layouts

#### Experimental Design

The growth chamber trial was set up in a completely randomized design with five repetitions of each treatment, and pots were rearranged during the experiment to minimize border effects.

All field trials were set up in randomized complete block designs with 3 blocks. Each block contained a plot with each studied variety, and each plot contained five replicate plants for each leaf harvest intensity treatment including controls.

#### Harvest Intensity and Frequency

Plants were defoliated at intensity levels of 0, 25, 50, 75, and 100% leaf removal.

In the growth chamber trials, the five harvest intensity treatments were applied one time (1×) at the 14 leaf stage [45 days after sowing (DAS)] to half of the plants, and three consecutive times (3×) at the 10, 14, and 18 leaf stages (35 DAS, 50 DAS, and 65 DAS, respectively) to half of the plants. In both cases, five plants were assigned to each treatment. Separate sets of control plants were used for the 1× trial and the 3× trial.

In the Danish field trials, the five harvest intensity treatments were applied one time (1×) at the 14 leaf stage (42 DAS in 2016 and 45 DAS in 2017), and two consecutive times (2×) at the 10 and 14 leaf stages (31 DAS and 42 DAS in 2016 and 39 DAS and 45 DAS in 2017). A third harvest, planned for the 18 leaf stage (to mirror the growth chamber trials) was canceled due to the initiation of flowering. For both harvest frequencies, five plants were sampled for each harvest intensity and harvest frequency per block.

In the 2016 Mexican field trial, the five harvest intensity treatments were applied two times (2×) at the 8 and 14 leaf stages (35 DAE and 50 DAE) and three times (3×) at the 8, 14, and 20 leaf stages (35 DAE, 50 DAE, and 65 DAE). Plants had begun to flower at the 16 leaf stage, so this trial allowed us to see the results of post-vegetative defoliation.

In the field experiments, five uniform plants per plot were randomly assigned to each defoliation intensity treatment by attaching tags to the base of their stems on the day of the first harvest. 1× and 3× frequency treatments were assigned to separate plots, which were each repeated three times in the block design. Each individual plot contained its own control plants (0% defoliation). In the growth chamber, all plants included in the experiment were assigned to one of the five defoliation intensity level treatments using tags, and 1× and 3× treatment plants were randomly dispersed throughout the same chamber. Leaves were systematically removed in equal proportion from the top to the bottom of the plant, to ensure a relatively equal balance of source and sink tissue removal. Defoliation was done with a scalpel from the base of the petiole.

### Plant Material

To align with the production systems of the Mexican farmers who initiated these experiments, we included an amaranth variety commonly used in the Oaxaca region of Mexico. *Amaranthus cruentus* var. Benito seeds were chosen after consulting Dr. Eduardo Espitia Rangel from experimental station CIR Centro INIFAP to ensure that the accession corresponded with the material used by Mexican farmers in Oaxaca. To test our hypothesis about widespread tolerance to defoliation across various genotypes under field conditions, we included six varieties adapted to the Danish climate, which represented the three primary grain amaranth species (Table 3). The variety used in the Mexican trial was selected based on suitability for the land chosen for the trial.

**TABLE 3:** Characteristics of varieties used across the leaf harvest trials.

**Name**	**Species**	**Source**	**Flower color**	**Seed color**	**Growth chamber**	**1× DK**	**2× DK**	**2× and 3× MX**
Benito	*A. cruentus*	MX^†^	Dark orange	Cream	×	×	×	
Françoise	*A. cruentus*	DK^*^	Light yellow	Pale cream				
Cecilia	*A. caudatus*	DK^*^	Pink	White + pink		×		
Inessa	*A. hypocondriacus*	DK^*^	Dark red	Cream		×		
Katia	*A. hypocondriacus*	DK^*^	Dark pink	Black		×		
Maria	*A. hypocondriacus*	DK^*^	Dark pink	Black		×	×	
Dorada	*A. cruentus*	MX^*w*^	Orange	Cream				×

^†^Indicates seeds from the USDA-ARS North Central Plant Regional Plant Introduction Station in Ames, Iowa. ^*^Indicates seeds from the University of Copenhagen’s breeding collection. ^*w*^Indicates seeds from Puente a la Salud Comunitaria’s seed bank.

### Measurements and Sample Preparation

#### Production Variables: Yield Components, Height, and Biomass

Following maturation, plant height was measured, then plants were dried until a stable dry-weight was reached (in a 70°C oven for the growth chamber trials, and at room temperature with fans for all field trials). Plant dry weight was taken, and the dry weight of previously harvested leaves was added to the dry weight of each mature plant to account for total harvested aboveground biomass. Total seed yield and 1000-KW were also measured. Harvest Index was calculated by dividing the seed yield by the total dry weight.

#### Nitrogen and Protein Determination

Seeds from the growth chamber trial and 2016 Danish field trial were ground into a fine powder using a Foss cyclotec 1093. Leaves that were harvested from the 2015 growth chamber trial were immediately frozen in liquid nitrogen and stored in a −80°C freezer. When ready for nitrogen analysis, each sample was ground using a mortar and pestle under liquid nitrogen. Nitrogen content (%N) was determined using gas chromatography with the organic elemental organizer “Flash 2000” from Thermo Scientific (Thermo Fisher Scientific Inc., 2008), which operates according to the dynamic flash combustion method (modified from the Dumas method). With this method, approximately 5 g of each sample were placed in tin capsules and an exact measurement (using three decimal places) was taken. The capsules were organized in a tray and placed into an oxidation/reduction reactor inside the elemental organizer machine. The temperature in the machine was increased to 900–1000°C. The machine delivered oxygen to the reactor, and the reaction between the oxygen and the tin capsule created an exothermic reaction, raising the temperature to 1800°C for a few seconds. Organic and inorganic substances were converted into elemental gasses, which were reduced and separated into a chromatographic column and detected by a thermal conductivity detector (Thermo Fisher Scientific Inc., 2008).

For the growth chamber trial, 5 g of seed from each plant were analyzed, as well as 5 g of leaves from each plant with each consecutive harvest. For the field trials, seeds from the five sampled plants/treatment/block were combined and three replicates of 5 g were prepared resulting in a total of nine measurements for each treatment. The percentage of nitrogen in the leaves measured in the laboratory was multiplied by the Jones Factor (6.25) to estimate total protein (Mariotti et al., 2008). This conversion factor is imperfect, but no conversion factor has been specified for amaranth by the Codex Alimentarius international food standards, and we were more interested in protein content differences between treatments rather than a precise measure of total protein. Because treatment had no impact on seed protein in the growth chamber and in 2016 Danish field trial, this analysis was not repeated in the other trials in order to reduce costs.

#### Canopy Light Interception

During the 2017 Danish 1× field trial, canopy light interception was measured on July 17th, 18 days after the 1× defoliation treatment. Since defoliated plants were mixed throughout the canopy, and the majority of plants in each plot were not subject to a defoliation treatment, we took multiple measurements throughout each plot to get an estimate of average canopy cover throughout. Photosynthetically Active Radiation (PAR) was measured using a 1 m linear ceptometer (Cavadevices, Buenos Aires, Argentina) between 11:30 and 14:00 h on a clear day. PAR was measured both above the canopy, then below the canopy at soil level in four different locations in each plot, and again above the canopy. The percentage of light infiltration below the canopy was calculated as 1 – (average PAR below/average PAR above) for each plot to determine whether canopy light interception impacted defoliation tolerance. Light infiltration was then plotted against seed yield ratios comparing control plant seed yield to average seed yield for each defoliation treatment in each plot to determine whether canopy light interception could predict the seed yield ratio.

### Data Analysis

All analyses were done in R version 00.99.484 (R Core Team, 2015).

For the growth chamber trial, analysis of variance (ANOVA) was used to test the effects of harvest treatments on each variable. Model residuals were tested for normality using Shapiro’s test and visual assessment of QQ residual plots. Tukey tests were then used for mean separation. Plants that did not produce flowers and seed were removed from seed yield, 1000 kernel weight, and seed protein models. For repeated measures (leaf biomass recovery), mixed models were built using the lme4 package in R. To test interactions between time and treatment, the data was organized into four separate binary matrices, one for each level of harvest above 0% (one matrix for 25% leaf harvest, one for 50%, etc.). The amount of biomass harvested between the first, second, and third harvest could then be compared at each harvest level individually.

For the field trials, the function lmer from the package lme4 was used to develop linear mixed-effects models (Bates et al., 2015). This allowed us to nest block effects within year effects, and treat both block and year as random effects. The random effects were calculated using ordinary maximum likelihood. Interactive and additive models for the fixed effects were tested using chi-square tests and plots of model residuals. Additive models were chosen where no significant interactions were observed. Tukey tests were then used for mean separation on the chosen model. In cases where the interactive model best described the data, binary model matrices were built to define linear hypotheses and compare unique combinations of treatments and varieties using the standard Tukey honest significance difference test. In all cases, a *p*-value of ≤0.05 was considered significant.

In the Mexican field trial there was a large difference in control plant seed yield in the 2× and 3× trials. As such, we ran an additional analysis of variance to test the impact of block on seed yield.

## Results

### Responses to Defoliation Under Controlled Conditions

**FIGURE 1:**
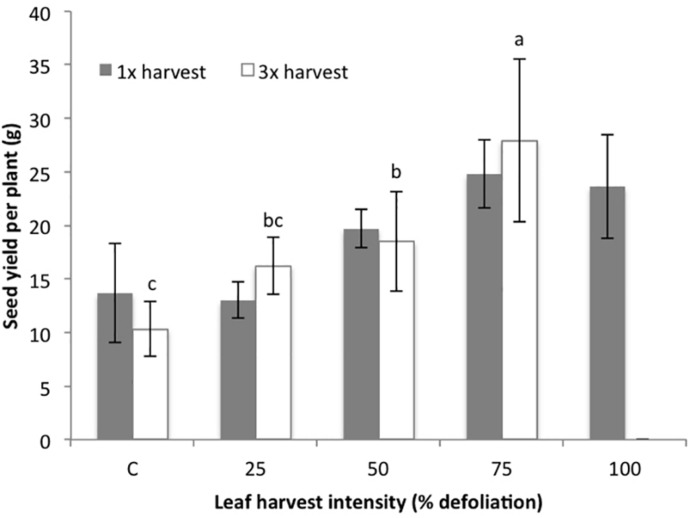
Seed yield (g/plant) in the growth chamber (2015) following either one (1×) or three (3×) consecutive harvests at five defoliation intensity levels. Separate control plants were used for the 1× and 3× studies, though the experiments were completed in the same growth chamber. Bars correspond to standard error. Lowercase letters correspond to the significance groups for 3× harvested plants (*p* ≤ 0.05, Tukey test). 1× treatments did not result in significant yield differences, so significance groups are not included.

Results show that leaf harvest intensity did not significantly impact seed yield (*p* = 0.111) at any level (25–100%) when leaves were harvested only once (1×) in growth chamber conditions. There appeared to be a slight, non-significant trend for seed yield to increase with harvest intensity following a single defoliation (Figure 1). However, following three harvests (3×), defoliation intensity significantly impacted seed yield (*p* = 0.0001). Many of the thrice defoliated plants did not flower, especially at higher harvest intensities. While all of the control and 25% defoliated plants flowered, only four out of five plants in both the 50 and 75% defoliation treatments flowered. In the 100% defoliation treatment, no plants flowered after three harvests. Despite fewer plants producing flowers, the 75% treatment had the highest average seed yield, followed by 50%, 25% and then control, even when including zero-values from non-flowering plants (Figure 1). While fewer plants flowered, those that did produced more seed overall. Thus, while there is a risk that plants will not flower following excessive leaf harvest, multiple harvests of up to 75% defoliation actually had a slightly (though not significant) positive effect on seed yield.

Seed size estimated by the 1000 Kernel Weight (1000 KW) seemed significantly impacted by 1× harvest (*p* = 0.0522), but no differences between treatment groups were identified when analyzed with Tukey. There were no differences in 1000 KW between 3× harvest treatments (*p* = 0.153). Despite the significance for the 1× treatment, there was no clear pattern or trend (Table 4). Harvest Index (HI) was not impacted following 1× harvest at any intensity level (*p* = 0.197), but following three harvests (3×), harvest intensity treatment did have a significant impact on HI (*p* = 0.00796). HI increased with each defoliation intensity level, but dropped off after 75% since none of the 100% treatment plants produced seeds (Table 4). Height and biomass each increased with higher defoliation intensities when defoliation occurred once (1×), and remained stable up to 75% after three defoliation events (3×) (Table 4). These results suggest that defoliation can actually boost both seed yield and vegetative biomass production under growth chamber conditions as long as defoliation occurs only once at any level, or multiple times up to a point (in this case up to 75% defoliation).

**TABLE 4:** Thousand Kernel Weight (KW), Harvest Index (HI), height, and total dry biomass measured at plant maturity after one leaf harvest event (1×) and three consecutive harvest events (3×) at five intensity levels (0, 25, 50, 75, and 100% leaf harvest) in 2015 growth chamber conditions.

**Leaf harvest intensity**	**1000 KW (g)**		**HI**		**Height (cm)**		**Final Biomass (g)**	
	**Mean**	**SE**	**Mean**	**SE**	**Mean**	**SE**	**Mean**	**SE**
**1× harvest**								
0%	0.78	0.01	0.14	0.02	151^B^	4.16	90.04	14.94
25%	0.75	0.01	0.14	0.01	150.4^B^	6.33	97.67	15.59
50%	0.80	0.01	0.16	0	161.8^AB^	2.17	99.46	8.36
75%	0.80	0.02	0.19	0.01	165.8^AB^	3.27	107.2	8.69
100%	0.81	0.02	0.14	0.02	184.3^A^	7.32	128.72	15.36
**3× harvest**								
0%	0.80	0.02	0.11^C^	0.01	148^A^	4.76	89	10.59
25%	0.77	0.01	0.15^BC^	0.01	16	2.47	112	9.62
50%	0.73	0.02	0.18^B^	0.05	160	3.16	126	8.26
75%	0.78	0.02	0.26^A^	0.07	160	16.0	109	21.26
100%	0^*^	0	0^*^	0	65	17.2	14	2.49

Letters represent significant groups (*p* ≤ 0.05, Tukey test). 0^*^Indicates that that the plants did not produce seed, and thus these samples were not included in the analysis. Letters correspond to significance groups (*p* ≤ 0.05, Tukey test). Where letters are not provided, the model was not significant at the 95% confidence interval.

**FIGURE 2:**
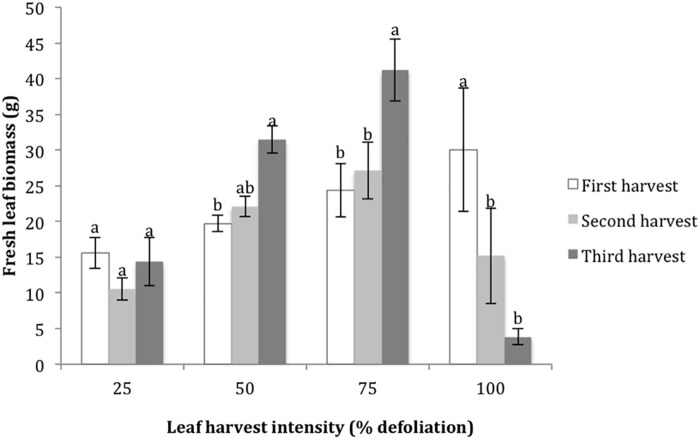
Harvested leaf biomass at four leaf harvest intensity levels during three consecutive defoliation events in the 2015 growth chamber trial. Bars represent standard error. Lowercase letters indicate significance groups for harvested leaf biomass within each harvest intensity level (*p* ≤ 0.05, Tukey test).

**FIGURE 3:**
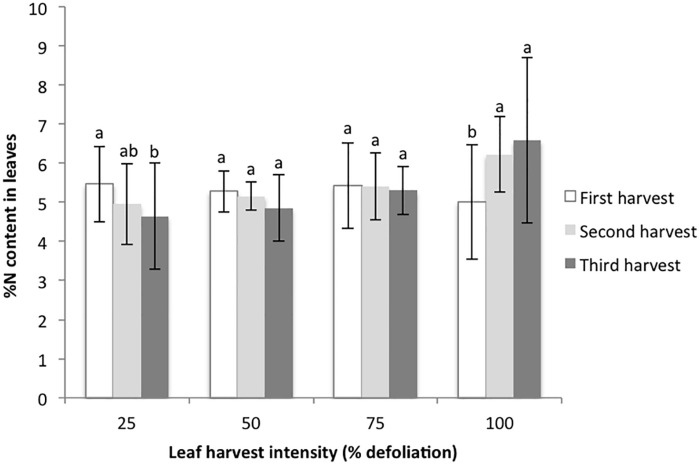
%N content in harvested amaranth leaves following three consecutive defoliation events in the 2015 growth chamber trial. Bars represent standard error. Lowercase letters indicate significance groups for %N content within each harvest intensity level (*p* ≤ 0.05, Tukey test).

The final metrics studied in the growth chamber were leaf biomass recovery following multiple defoliations, and % nitrogen in leaves with each harvest. For the 25% harvest treatment, the amount of leaf biomass harvested was not significantly different with each successive harvest. For the 50 and 75% harvest treatments, the amount of harvested leaf biomass increased with each successive harvest, suggesting that higher levels of harvest stimulated new biomass production (Figure 2). In contrast, 100% defoliation resulted in significant leaf biomass reductions with each successive harvest (Figure 2). The distribution of residuals for the leaf biomass model was non-normal, even with transformation through Box Cox methods. This was likely due to the very low biomass values for the 100% treatment at the third harvest. %N decreased in leaves with each successive harvest at 25% defoliation, remained stable at 50 and 75%, and increased at 100% (Figure 3).

### Responses to Defoliation Under Field Conditions: One-Time Harvest (1×) Results

Across both years (2016 and 2017) of Danish field trials, leaf harvest intensity and variety significantly impacted seed yield (*p* < 0.0001 and *p* < 0.0001, respectively). Leaf harvest intensity and variety did not interact (*p* = 0.355). For all varieties, seed yield was not significantly reduced by a single defoliation event with 25 or 50% defoliation at the 14-leaf stage approximately halfway through vegetative development (Table 5). At 75% defoliation, seed yield reductions were significant compared to lower intensity treatments. At 100% defoliation, seed yield reductions were significant compared to all treatments (Table 5). While seed yield was much higher overall in 2016 compared to 2017, seed yield reductions relative to controls followed the same pattern each year (Table 5). This demonstrates a consistent pattern of stable seed production with a single 25–50% defoliation event, and detrimental impacts to seed yield with a single 75–100% defoliation event.

**TABLE 5:** Seed yield (g/plant) and standard error (SE) for all varieties studied in Danish field conditions in 2016 and 2017 under the 1× harvest frequency treatment.

**Leaf harvest intensity**	***A. cruentus***				***A. caudatus***		***A. hypocondriacus***						
	**Benito**		**Françoise**		**Cecilia**		**Inessa**		**Katia**		**Maria**		**Sig**
	**Mean**	**SE**	**Mean**	**SE**	**Mean**	**SE**	**Mean**	**SE**	**Mean**	**SE**	**Mean**	**SE**	
**2016 yield**													
0%	27.44	1.31	33.86	6.48	36.71	3.94	21.34	1.24	34.32	5.89	32.51	3.76	*a*
25%	17.25	3.89	24.73	5.00	31.85	4.04	22.00	0.27	31.09	6.22	21.29	4.27	*a*
50%	18.04	6.69	27.55	7.82	32.98	3.58	19.73	2.84	33.50	8.42	22.37	5.28	*a*
75%	12.73	5.51	9.17	0.83	17.93	2.74	12.93	1.63	29.66	2.53	14.85	0.82	*b*
100%	2.39	0.77	4.25	0.58	10.26	0.62	4.66	2.40	9.74	1.85	9.40	2.41	*c*
**2017 yield**													
0%	9.04	1.08	6.17	0.94	3.16	1.45	8.95	1.75	8.92	1.51	7.92	2.37	*a*
25%	12.26	2.39	9.05	3.18	3.85	0.22	7.63	1.58	10.32	2.23	8.39	0.91	*a*
50%	9.71	1.61	5.08	2.14	3.62	0.24	8.77	1.53	9.24	0.90	7.47	1.47	*a*
75%	5.73	1.39	6.32	1.03	3.62	0.24	8.03	1.16	7.87	1.02	6.00	0.96	*b*
100%	2.56	0.51	2.57	0.81	1.00	0.33	2.18	1.14	3.81	0.87	3.22	0.28	*c*

*Letters in the Sig (significance) column indicate significance groups (*p* ≤ 0.05, Tukey test). As there were no interactions between harvest intensity treatments and variety, groups are reported for all varieties. Gray shading is used to distinguish species groups. A. cruentus varieties include Benito and Francoise, A. caudatus groups include Cecilia, and A. hypocondriacus groups include Inessa, Katia, and Maria.*

Total plant biomass at maturity was significantly impacted by defoliation intensity treatment (*p* = 5.54e-10). Tukey tests revealed that 100% defoliation resulted in smaller plants compared to all other treatments. Control plants were larger than plants undergoing 75% defoliation (*p* = 0.002), as were 25% defoliation treated plants (*p* = 0.057). Harvest Index was not impacted by defoliation (*p* = 0.1024), nor was 1000 KW (*p* = 0.6545).

**FIGURE 4:**
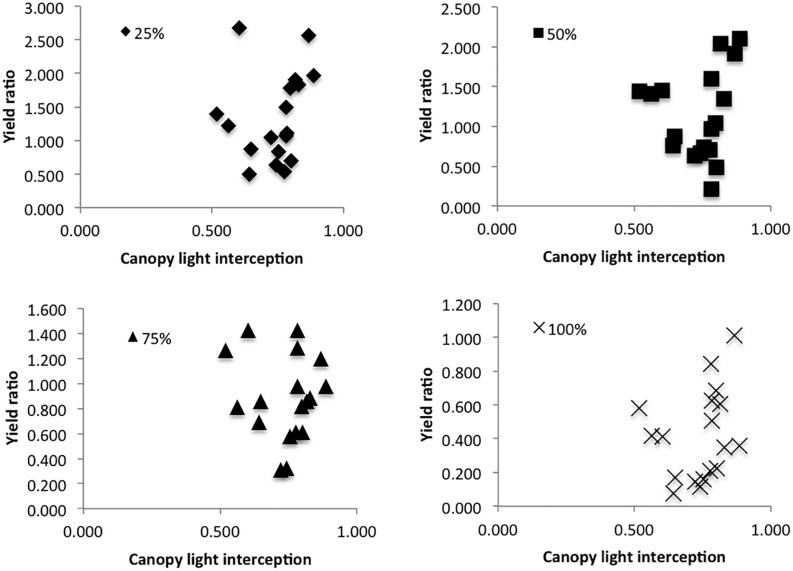
Yield ratio (average seed yield from each plot per treatment/average seed yield of control plants in the same plot), as predicted by canopy light interception (1 – PAR above the canopy/PAR below the canopy).

In the 2017 trial, canopy light interception was plotted against % seed yield compared to control for each treatment; canopy light interception did not seem to have an effect on defoliation tolerance (Figure 4).

### Responses to Defoliation in Danish Field Conditions: Consecutive Harvest (2×) Results

Across both years of the 2× defoliation study in Danish field conditions, *A. cruentus* var. Benito seed yield was stable up to 50% defoliation. In 2016, there was a non-significant trend for seed yield to increase with 25 and 50% defoliation compared to controls (Table 6). Because the Maria variety was only studied one of the 2 years, it was analyzed separately from the Benito data. *A. hypocondriacus* var. Maria plants had stable seed yield following two consecutive leaf harvests (2×) at the level of 25% defoliation, but seed yield dropped significantly after 50% (Table 6).

**TABLE 6:** Seed yield (g/plant), biomass, and Harvest Index (HI) with standard error (SE) for two consecutive (2×) leaf harvest events in Danish field conditions during 2016 and 2017.

**Leaf harvest intensity**	**Benito (*A. cruentus*)**						**Maria (*A. hypocondriacus*)**					
	**Yield**	**SE**	**Biomass**	**SE**	**HI**	**SE**	**Yield**	**SE**	**Biomass**	**SE**	**HI**	**SE**
**2016**												
0%	20.06^A^	3.88	112.47^A^	17.15	0.18^A^	0.01						
25%	27.06^A^	7.45	149.65^A^	38.08	0.18^A^	0.01						
50%	23.95^A^	2.36	134.21^A^	9.47	0.18^A^	0.01						
75%	8.52^B^	1.28	44.45^B^	4.42	0.18^A^	0.01						
100%	0.88^B^	0.41	9.96^B^	2.52	0.08^B^	0.03						
**2017**												
0%	11.10^A^	2.56	59.15^A^	13.55	0.19^A^	0.01	8.83^A^	0.88	33.97^A^	2.94	0.26^A^	0.01
25%	11.02^A^	2.81	51.39^A^	11.25	0.21^A^	0.01	7.39^AB^	0.67	29.99^A^	1.88	0.25^A^	0.01
50%	8.17^A^	0.99	40.00^A^	4.54	0.20^A^	0	5.51^BC^	0.76	23.08^B^	2.01	0.24^A^	0.01
75%	4.91^B^	1.2	25.01^B^	4.74	0.19^A^	0.02	4.02^C^	0.96	17.72^B^	2.51	0.22^A^	0.02
100%	0.94^B^	0.14	7.72^B^	0.6	0.12^B^	0.01	1.28^D^	0.3	7.4^C^	0.83	0.17^B^	0.02

*Letters represent significant groups (*p* ≤ 0.05, Tukey test).*

Total plant biomass at maturity was significantly impacted by multiple defoliation events for both the *A. cruentus* Benito plants (*p* < 0.0001) and the *A. hypocondriacus* Maria plants (*p* < 0.0001). In both cases, the 75 and 100% treatments resulted in significantly smaller plants than the three other treatments (Table 6).

Harvest Index was significantly impacted by harvest intensity in the 2× trial for both Benito (*p* < 0.0001) and Maria (*p* < 0.0001), with stable HIs until 100%, at which point the HI dropped significantly in all cases (Table 6).

In the Maria plants, defoliation had a significant effect on 1000 KW (*p* < 0.0001). However, the only treatments in the 2× trials that resulted in significantly different 1000 KWs from one another were the 50 and 100% treatments (*p* = 0.0063). For Benito plants, harvest intensity treatments appeared to significantly impact 1000 KW (*p* = 0.0007), however, when Tukey tests were used, no treatment was significantly different from another (results not shown).

### Responses to Defoliation in Mexican Field Conditions: Consecutive Harvest (2× and 3×) Results

For both the 2× and 3× trials, harvest intensity had a significant effect on seed yield (*p* < 0.0001 for both). Compared to control plants, 25 and 50% defoliation did not significantly reduce seed yield (Table 7). At 50% defoliation there was a statistically non-significant reduction in seed yield for both the 2× and 3× treatments (Table 7). Around 75 and 100% defoliation resulted in significant seed yield declines (Table 7). Controls were repeated in each plot, and thus the average seed yield for control differs between the 2× and the 3× plots. Results from the analysis of variance to determine block effects showed that block was a significant predictor of seed yield at the 10% level (*p* = 0.0992), and block and treatment did not interact (*p* = 0.1715). Despite differences in control seed yield, patterns in seed yield tolerance at different levels of defoliation were similar to Danish results.

**TABLE 7:** Seed yield (g/plant) and standard error (SE) results from Mexican field conditions (2016) following two (2×) and three (3×) consecutive leaf harvest events at five intensity levels.

**Leaf harvest intensity**	**3×**		**2×**	
	**Yield**	**SE**	**Yield**	**SE**
0%	27.21^A^	3.66	40.33^A^	6.51
25%	25.80^A^	3.50	39.65^A^	5.52
50%	18.73^AB^	2.74	26.64^AB^	3.51
75%	11.68^BC^	1.99	25.45^AB^	5.05
100%	7.17^C^	2.18	9.93^B^	2.01

*Letters indicate significance groups (*p* ≤ 0.05, Tukey test).*

### Seed Protein Impacts From 2016 Trials

In the growth chamber trials, seed protein content (measured in %N ^*^ 6.25) was not significantly impacted by leaf harvest at any intensity level for either the 1× (*p* = 0.737) or 3× harvest trials (*p* = 0.691).

In the 1× Danish field trial (2016), seed protein was significantly impacted by leaf harvest intensity and variety, with a significant interaction between the two studied factors (*p* = 0.0003). Tukey tests were done on each unique combination of variety and harvest intensity, and showed that the leaf harvest intensity level only affected the seed protein content of varieties Maria and Katia, without a clear response pattern as harvest intensity increased (Table 8). As such, protein results did not show conclusive trends, and did not suggest significant variation between treatments. Therefore, further protein analysis was not pursued.

**TABLE 8:** Crude seed protein content in seeds (%N ^*^ 6.25) and standard error (SE) in six amaranth varieties subjected to one defoliation event (1×) at five harvest intensity levels from Danish field trials in 2016.

**Leaf harvest intensity**	***A. cruentus***				***A. caudatus***		***A. hypocondriacus***					
	**Benito**		**Françoise**		**Cecilia**		**Inessa**		**Katia**		**Maria**	
	**Yield**	**SE**	**Yield**	**SE**	**Yield**	**SE**	**Yield**	**SE**	**Yield**	**SE**	**Yield**	**SE**
0%	12.64^A^	0.25	12.87^A^	0.21	13.38^A^	0.08	13.48^A^	0.24	13.22^A^	0.14	12.15^AB^	0.23
25%	12.99^A^	0.18	12.87^A^	0.08	12.64^A^	0.23	12.26^A^	0.23	13.10^AB^	0.30	12.33^AB^	0.16
50%	13.1^A^	0.10	13.01^A^	0.14	13.39^A^	0.30	13.21^A^	0.11	12.21^B^	0.18	12.48^AB^	0.43
75%	12.8^A^	0.24	12.65^A^	0.21	13.53^A^	0.17	14.74^A^	1.02	12.21^B^	0.40	11.59^A^	0.38
100%	12.62^A^	0.25	12.88^A^	0.26	13.44^A^	0.15	14.08^A^	0.28	13.76^A^	0.23	12.90^B^	0.43

*Letters represent significance groups of harvest intensity treatments within each variety (*p* ≤ 0.05, Tukey test).*

## Discussion

### Amaranth Tolerance to Defoliation Is Widespread Across Cultivars and Environments

Amaranth is grown and consumed by small-scale farmers around the world, many of whom face nutritional and financial insecurity (Rastogi and Shukla, 2013). Prior to beginning this study, the primary author spent time with amaranth farmers in Oaxaca, Mexico, who expressed interest in establishing baseline recommendations for using amaranth plants for both leaves and seeds. This study was developed around this practical goal, and built upon the results of others who have studied leaf harvest dynamics in amaranth. While other studies have identified genetic differences in tolerance between cultivars at high levels of defoliation (100%), we aimed to determine whether at more moderate levels, we could identify similarities in defoliation tolerance across cultivars and climates. We also aimed to better understand the relationship between harvest frequency and intensity in determining defoliation tolerance.

Overall, with a single defoliation event, we found that amaranth seed yield was not significantly affected by up to 50% defoliation in any of the three distinct environments studied (growth chamber, Danish field conditions, and Mexican field conditions in Oaxaca). This remained true across the three studied species (*A. cruentus*, *A. hypochondriacus*, and *A. caudatus*) and the seven studied cultivars. However, in Mexican conditions and in some of the Danish varieties in 2016, seed yield was slightly lower following 50% defoliation (though not significantly: all *p*-values were above 0.05) than control and 25% plants. At 25% defoliation, we did not see negative seed yield effects in any environment or variety studied. For some varieties in Denmark in both the growth chamber and field trials, 25% defoliation mid-way through vegetative development actually boosted seed yield. This was true for 5 of the 6 varieties tested in 2016, and 1 of the 6 varieties in 2017. Defoliation did not result in changes to seed quality, as measured by seed protein and 1000 KW.

A plant’s defoliation tolerance and ability to relocate carbon and nitrogen is heavily influenced by its evolutionary history and the need to adapt to herbivory (Stowe et al., 2000). For example, plants in populations that are frequently grazed or areas that are frequently mowed such as grass lots exhibit grater defoliation tolerance than plants of the same species in unmanaged areas like ditches (ibid). In the context of amaranth, *Amaranthus cruentus* and *Amaranthus hypocondriacus* cultivars from Mexico that have been used for dual-use purposes for centuries are likely to have adapted to defoliation. The fact that we observed similar yield responses in cultivars developed outside of this system is promising for amaranth farmers in other parts of the world who wish to adopt this practice. Despite the genetic differences identified for defoliation tolerance in Roitner-Schobesberger and Kaul (2013) and Dinssa et al. (2018), these differences did not appear significant when defoliation occurred at low to moderate levels (25–50% leaf removal). These results are encouraging, as they indicate that *Amaranthus* plants across species and originating from different parts of the world show potential to be used in dual-use vegetable/seed systems.

One 2012 study on amaranth defoliation showed that tolerance to defoliation decreased under drought stressed conditions (Castrillón-Arbeláez et al., 2012). However, even in Oaxaca’s much drier climate, amaranth was able to compensate for defoliation stress. That said, for producers in drought prone areas who wish to practice dual-use production of amaranth, it may be of benefit to use deeper-rooted varieties. One of the most common tolerance mechanisms used by plants is to mobilize stored carbon in the roots and partition it into source tissues (Stowe et al., 2000). A 2012 study on leaf removal in *Ruellia nudiflora* found that in this particular species, percentage of biomass allocated to roots prior to damage was directly related to compensatory ability. In other words, genotypes that invest more biomass into roots proportional to vegetative biomass exhibit improved ability to compensate for leaf damage (Rivera-Solís et al., 2012). Liu and Stützel (2004) assessed drought responses in four vegetable amaranth varieties. They found that following drought conditions, all four varieties increased their root to shoot dry mass ratios. Plants that started with greater root biomass had to compensate less for drought; they still decreased their shoot dry mass production, but they put less energy into root production than plants with less root biomass (Liu and Stützel, 2004). It is worth noting that while genotype is important in determining root to shoot ratios, differences in root biomass production are not entirely based on genotype. Strauss and Agrawal (1999) reported that high nutrient conditions tend to reduce the root to shoot ratio in plants, and thus high nutrient environments could actually make plants less tolerant to herbivory. Other experiments on nutrient availability and stress tolerance have produced variable results (Suwa and Maherali, 2008). While our results in Oaxaca’s dry climate were promising, we only studied one variety in this context. Based on the results of Castrillón-Arbeláez et al. (2012), we would suggest further variety trials before adopting dual-use systems in drought-prone areas.

### Comparisons Between Trials

Seed yield for control plants was substantially lower in the growth chamber trials than in Mexican or Danish field trials, with the exception of 2017 Danish trials (Figure 1 and Tables 5, 7). This is consistent with other studies; plants grown in growth chambers tend to be smaller overall with lower biomass and seed yield (Poorter et al., 2016). The light and temperature conditions used in our growth chamber study (reported above) were consistent with the “low to intermediate light levels” in growth chambers described by Poorter et al. (2016). These conditions tend to produce plants that are “source limited”; one might interpret this to mean that growth chamber-grown plants should be less tolerant to defoliation, given the importance of stored carbon in stem and root tissues in determining defoliation tolerance. However, while control plants in the growth chambers produced fewer seeds, these growth chamber-grown plants responded more positively to defoliation under growth chamber conditions than field conditions. This is consistent with the results presented by Vargas-Ortiz et al. (2013). The plants grown in the growth chamber experienced more constant growth conditions; they were watered at a consistent rate each day and did not experience daily variation in light and temperature. In field conditions, more variables (water, temperature, light, weed competition, more potential for more insect pest exposure) can add additional stressors that may impact seed yield and stress tolerance. Vargas-Ortiz et al. (2013) also noted that defoliated plants in field conditions were more susceptible to root rot pathogens than control plants.

Seed yield was substantially higher in the 2016 Danish field trials than the 2017 trials despite the similarity of environments. Due to heavy rains in the fall of 2017, harvest was delayed. This may have resulted in higher rates of seed shatter in 2017 as well as a reduced drying off period to allow proper seed set. This is consistent with the findings of Myers (1996), who described that amaranth does not have uniform seed development within the panicle, and that seed shatter can begin before all of the seeds in a panicle are mature, especially during periods of strong wind or rain. Indeed in September of 2017, Taastrup, Denmark experienced 130 mm more rainfall than in September of 2016 (Table 2). Additionally, plants were wet when harvested in 2017 and had to be dried indoors. Seed yield was also higher in the 2× 2016 Oaxaca trial than the 3× 2016 Oaxaca trial for control plants. The best explanation for this is random variation between plots, likely due to fertility differences. As these plots were managed manually without mechanization, it is reasonable to expect some differences between plots. The ANOVA analysis showed that block (plot) had a potentially significant effect (significant at the 10% level) on seed yield. However, each trial (2× and 3×) was repeated three times with control plants in each treatment, and the overall trends in defoliation tolerance remained consistent across plots.

### Shading and Leaf Biomass Dynamics

Several factors determine a plant’s ability to recover from defoliation. Carbon partitioning is complex; some factors that determine partitioning include the size and number of sink tissues, shading, vascular connections throughout the plant, growth rate, nutrient ability, temporary storage of carbon in leaves (Wardlaw, 1990). While a complete study of tolerance mechanisms was beyond the scope of this study, our results shed some light on leaf canopy dynamics.

Dinssa et al. (2018) found that amaranth plants with more leaves were more tolerant to defoliation. The leaf data from the growth chamber phase of our study builds upon this finding by showing that defoliation (up to 75% under controlled conditions) can actually stimulate leaf production. A 2013 study on defoliation in pumpkins showed that with increasing intensities of weekly leaf harvest, the plants with the highest levels of defoliation responded by producing the highest amounts leaf biomass (Isutsa and Mallowa, 2013). Even at the least intensive defoliation treatment in this study, plants were harvested each week for 21 and 29 weeks (season 1 and 2), with a minimum of around 400 and 200 leaves removed from each plant. These defoliation rates were high enough that they resulted in significant reductions in fruit production. However, the result that more defoliation led to increased leaf production can be connected to the results of Vargas-Ortiz et al. (2015), which showed that a plant’s ability to produce photosynthetic tissues following defoliation is directly linked to the plant’s defoliation tolerance. While there is a clear drop in seed yield following high levels of defoliation (in our case 75% or more in field conditions), the increase in leaf biomass following defoliation seems to be an important tolerance mechanism in amaranth, similar to the observations by Isutsa and Mallowa (2013) in pumpkins. At lower levels (25–50%), our growth chamber results suggest that defoliated amaranth plants will produce more leaf biomass than non-defoliated plants, and this overcompensation may be a mechanism for increased seed yield.

While leaf area determines the percentage of incident radiation that a plant is able to intercept, and thus its capacity for photosynthesis (Isutsa and Mallowa, 2013), this increased ability may be compromised by shading in the canopy. Vargas-Ortiz et al. (2013) found that when amaranth plants are heavily shaded, they undergo similar biochemical processes of carbon remobilization and enzyme activity as the processes they experience following defoliation. The combined effects of defoliation and shading are thus likely to create compounding stress effects on individual plants. Many studies have shown the importance of remobilization of carbon and nutrients from root and shoot tissues in determining defoliation tolerance (Mihaliak and Lincoln, 1989; Wardlaw, 1990; Vargas-Ortiz et al., 2013). However, new leaves must have access to adequate light in order to photosynthesize and produce the energy needed for recovery. In the 2015 growth chamber experiment 80% of plants in the chamber were defoliated to some degree (all but the control treatment), whereas in field conditions, we defoliated individual plants and left the plants around them untouched. As such, canopy shading in the growth chamber was significantly reduced with each defoliation event, potentially allowing for higher photosynthetic activity in leaves lower in the canopy. In the field, we defoliated (and thus reduced the photosynthetic capabilities of) individual plants without modifying the canopy shading. This could have created higher competition for light among defoliated plants in field conditions compared with defoliated plants in the growth chamber, even if they were able to increase leaf production following the defoliation event. Additionally, growing plants in pots tends to reduce plant-to-plant interactions, and thus shading effects tend to be reduced in growth chambers (Poorter et al., 2016). While we did see higher defoliation tolerance in the growth chamber, our 2017 Danish field trial results suggested that canopy cover did not have a significant effect on defoliation tolerance (Figure 4). Although our results suggest that canopy cover is not an important determinant of tolerance, a potentially interesting approach for future field trials might be to defoliate whole stands of plants rather than individual plants in a canopy in order to further evaluate the role of shading in determining defoliation tolerance.

A final factor we considered related to canopy and photosynthesis dynamics is that there is a strong correlation between photosynthetic capacity and leaf nitrogen. Plants that accumulate more nitrogen in their leaves are more likely to have higher rates of photosynthesis (Hikosaka, 2005). Our growth chamber results suggest that defoliation triggers nitrogen remobilization into leaves. At higher rates of defoliation, subsequently harvested leaves had higher rates of nitrogen content than leaves from plants with low levels of defoliation (Figure 3). Hikosaka (2005) studied this dynamic with shading and found that shading in the lower canopy results in nitrogen remobilization to new, sunlit leaves. While we have discussed the importance of stored nutrients and carbon in root and stem tissues, leaves are an important source tissue in amaranth, and their removal represents an important loss of stored assimilates (Roitner-Schobesberger and Kaul, 2013). Even so, our study shows that with the removal of some of these leaves (up to 75% in growth chamber conditions), and thus removal of an important nitrogen source, plants are able to utilize other nitrogen stores, likely from the soil, remaining leaves, and their root systems. These dynamics should be studied further under various fertilization regimes for more insight on these dynamics in field conditions.

### Multiple Low-Intensity Harvests Events Are Better Than One High-Intensity Harvest Event

Following multiple harvests (2× in Danish fields, 2× and 3× in Mexican fields, and 3× in the growth chamber), yields were stable across environments and genotypes at up to 25% defoliation. In all but the *A. hypocondriacus* var. Maria variety in Denmark, yields were actually stable with up to 50% defoliation on 2–3 consecutive occasions. More importantly, yields were comparable to 1× defoliated plants (e.g., comparing 25% defoliation 1× to 25% defoliation 2× or 3×). While many other studies have assessed tolerance to a single defoliation event, this is the first paper to compare the effects single defoliation events to consecutive defoliation events on the same cultivars and in the same environments. In all of our experiments where 1× and 2× or 3× defoliation were studied side by side, multiple low to moderate intensity defoliation events resulted in higher seed yield than single high intensity defoliation events. These results suggest that amaranth leaves could be harvested at low levels throughout vegetative development to provide household nutrition throughout the growing season without significantly damaging seed yield.

Mihaliak and Lincoln (1989) studied the impacts of continuous defoliation in *Heterotheca subaxillaris*. They removed every fourth or every other new leaf, and compared these treatments with control plants. At the end of the study, the lower level defoliation treatment resulted in a similar root to shoot ratio to that of control plants. Plants from the higher defoliation treatment reduced the root to shoot ratio (Mihaliak and Lincoln, 1989). Presumably, by removing fewer leaves, the plant requires less remobilization of carbon and nutrients from its stores in root and stem tissues. If we combine these results with the discussion in the Section “Shading and Leaf Biomass Dynamics” showing that leaf harvest can stimulate leaf production, we can hypothesize that low levels of defoliation can sufficiently stimulate leaf production and photosynthesis to quickly compensate for carbon and nutrient relocation from stored root and shoot sources. As long as the root to shoot ratio is not significantly disrupted, plants can retain yields. It should be noted that low soil fertility can negatively impact defoliation tolerance (Mihaliak and Lincoln, 1989), as can cool weather conditions (Vargas-Ortiz et al., 2015) and drought (Castrillón-Arbeláez et al., 2012). All of these factors may impact the ratio of stored carbon and nutrients in root and shoot tissues. More frequent but less intensive defoliation events may allow a plant to better cope with these additional stressors than one high-intensity defoliation event, but, extra caution should be practiced when using amaranth plants for dual use purposes in systems with these additional environmental stressors.

## Conclusion

The results of this study build on previous defoliation research in amaranth to show that tolerance to defoliation is widespread across environments and cultivars of various origins of the three primary grain amaranth species *A. cruentus*, *A. hypocondriacus*, and *A. caudatus*. Using amaranth as a dual-use crop for leafy green and seed production is a viable and promising opportunity for small-scale growers to support household nutrition from leaves as well as income from seeds. While other studies have identified genetic differences in defoliation tolerance at high levels of defoliation (100%), we found that at more moderate defoliation levels (25–50%), seed yield remained stable across cultivars and environments. Specifically, seed yield and quality remained stable for most cultivars in most environments following 1–3 defoliation events of up to 50% leaf removal, and for all cultivars in all environments following 1–3 defoliation events of 25%. We also identified that multiple low-intensity defoliation events (∼25% leaf removal) is preferable to a one-time high-intensity (75% or higher) defoliation event. These results support the idea that farmers across the world who wish to use amaranth plants for both leaves and seeds could do so at low to moderate levels (25–50%, up to three times) without compromising seed yield or quality.

## Author Contributions

NH developed the research hypothesis and protocols, conducted the trials, performed the experimental and statistical analysis, and wrote the manuscript. GA oversaw the project, provided the extensive editing and guidance, and assisted with experiments. MDG conducted the Mexican field trials and analyses. S-EJ provided the editing and feedback.

## Conflict of Interest Statement

The authors declare that the research was conducted in the absence of any commercial or financial relationships that could be construed as a potential conflict of interest.

## Footnotes

1 https://www.coneval.org.mx/Medicion/MP/Paginas/AE_pobreza_2016.aspx, data under “Anexo Estadístico” tab 4A

2 http://www.puentemexico.org/en/content/the-numbers
